# Cutaneous Side Effects of BRAF Inhibitors in Advanced Melanoma: Review of the Literature

**DOI:** 10.1155/2016/5361569

**Published:** 2016-03-03

**Authors:** Bilgen Gençler, Müzeyyen Gönül

**Affiliations:** Department of Dermatology, Ministry of Health Diskapi Yildirim Beyazit Education and Research Hospital, 06110 Ankara, Turkey

## Abstract

The incidence of melanoma has recently been increasing. BRAF mutations have been found in 40–60% of melanomas. The increased activity of BRAF V600E leads to the activation of downstream signaling through the mitogen-activated protein kinase (MAPK) pathway, which plays a key role as a regulator of cell growth, differentiation, and survival. The use of BRAF inhibitors in metastatic melanoma with BRAF mutation ensures clinical improvement of the disease. Vemurafenib and dabrafenib are two selective BRAF inhibitors approved by the Food and Drug Administration (FDA). Both drugs are well tolerated and successfully used in clinical practice. However, some adverse reactions have been reported in patients in the course of treatment. Cutaneous side effects are the most common adverse events among them with a broad spectrum. Both the case reports and several original clinical trials reported cutaneous reactions during the treatment with BRAF inhibitors. In this review, the common cutaneous side effects of BRAF inhibitors in the treatment of metastatic melanoma with BRAF V600E mutation were reviewed.

## 1. Introduction

Melanoma is a lethal type of skin cancer that is derived from melanocytes. The incidence of melanoma has been increasing in recent decades and the mortality is approximately 10% [1]. Although the patients with early stage melanoma can be cured with surgery, the prognosis of patients with inoperable metastatic melanoma is poor, with a 5-year survival rate of <10% and a median survival of <1 year [2].

Until recently, dacarbazine and interleukin-2 were the only agents approved by the Food and Drug Administration (FDA) for the standard treatment of metastatic melanoma [3]. The clinical development of targeted therapies of mitogen-activated protein kinase (MAPK) pathway is a milestone in the management of advanced melanoma.

## 2. MAPK Pathway

The mitogen-activated protein kinase (MAPK) is an important signaling pathway that plays a key role as a regulator of cell growth, differentiation, and survival. When an extracellular ligand binds to specific plasma membrane receptor tyrosine kinase, a series of phosphorylation including RAS, RAF, MEK, and ERK mediates the growth signals to the nucleus to promote cell proliferation, differentiation, and survival [4].

The mutation of the MAPK pathway is the critical point at the pathogenesis of melanoma. BRAF mutations were found in approximately 40–60% of cutaneous melanomas and V600E is the most common type of these mutations. It was shown that valine is substituted by glutamic acid at position 600 in 90% of BRAF mutant melanomas [5, 6]. The increased activity of BRAF V600E leads to the activation of downstream signaling through the MAPK pathway. Constitutive oncogenic signaling causes apoptosis prevention and excessive cell proliferation [6]. Additionally, BRAF mutations were associated with a poor prognosis in patients with metastatic disease [7].

## 3. BRAF Inhibitors

After the discovery of BRAF mutations, clinical trials of targeted therapies of advanced melanoma show significant improvement. The selective inhibitors of mutant BRAF kinase have become the key component of the treatment of metastatic disease. Vemurafenib and dabrafenib are two BRAF inhibitors (BRAFi) that have been licensed by FDA for the treatment of metastatic melanoma with mutant BRAF V600 [2].

Vemurafenib was the first selective tyrosine kinase inhibitor that demonstrated antitumor activity by blocking the activation of MAPK kinase pathway [2]. The antitumor activity of vemurafenib was observed in melanoma cell lines with BRAF V600E mutation, but not in wild-type melanomas [8]. Dabrafenib was the second reversible and potent selective inhibitor of BRAF V600 kinase approved by the FDA [2]. The use of BRAFi significantly increases the response rate, and prolonged progression-free and overall survival in melanoma patients with BRAF mutation [8, 9]. These oral agents are well tolerated, but some adverse events can occur due to paradoxical reactivation of MAPK signaling [10]. This review aimed to determine the most common cutaneous side effects due to BRAFi that has been used in advanced melanomas.

## 4. Cutaneous Side Effects

Dermatologic reactions related to the treatment of BRAFi in advanced melanoma are well known common side effects. The rate of cutaneous adverse events associated with vemurafenib was 92% to 95% of patients in the BRAF inhibitor melanoma (BRIM) studies [11]. However, the cutaneous adverse events related to dabrafenib in BREAK studies were similar to those due to vemurafenib in BRIM studies, and the percentages of these side effects varied in both of the studies [9, 12, 13]. Skin reactions usually occur within days of undergoing treatment. Adverse events (AEs) can be classified in five grades: grades 1-2 as mild to moderate, grade 3 as severe adverse event, grade 4 as life-threatening adverse event, and grade 5 as fatal adverse event [14]. The most seen adverse events previously reported were grade 1 or 2, so patients could continue treatment without dose modifications [11]. Percentages of common (>5%) cutaneous adverse events with vemurafenib and dabrafenib treatment are shown in Tables 1 and 2.

### 4.1. Inflammatory Dermatoses

Skin rash was one of the most commonly reported AEs [11]. The incidence of skin rash was reported as 75% in the BRIM 2 study and 64% in the BRIM 3 study, mostly with grade 1 or 2 severity [6, 8, 11]. It was revealed in the BRIM 3 study that 10% of patients developed grade 2 skin rash and 8% of patients developed grade 3 skin rash [8]. Skin rash usually occurred on face, neck, trunk, and extremities and appeared with a mean time of 1.6 weeks after vemurafenib treatment. Many subtypes of rash were seen, but the most common was not otherwise specified with a range of 37%–54%. Maculopapular rash due to vemurafenib therapy was another clinical feature and linked to a hypersensitivity reaction. Serious cutaneous adverse events such as Stevens-Johnson syndrome, toxic epidermal necrolysis, cellulitis, drug reaction with eosinophilia, and systemic symptoms (DRESS) were rarely seen but could cause drug discontinuation [5, 11, 15, 16].

Boussemart et al. reported grade 1 xerosis in 33% of patients with a median time of 57 days. Xerosis could provoke mild pruritus [17]. Pruritus was observed in patients treated with vemurafenib with an incidence of 29% in the BRIM 2 study [6]. Six percent of patients with grade 2 and 1% of patients with grade 3 pruritus were reported in the BRIM 3 study [8]. In the BREAK-2 study with dabrafenib, 10% of patients experienced pruritus, while Sanlorenzo et al. reported pruritus both in vemurafenib and in dabrafenib treatment groups with a percentage of 33.3% [13, 14].

Anforth et al. reported acneiform eruptions in 3% of patients who were treated with BRAFi longer than 52 weeks. These lesions were seen at areas such as face, trunk, and upper limbs [19]. The incidence of follicular papulopustular rash was 6% in the study by Mattei et al. [18].

Boussemart et al. described grade 1 or 2 erythematous hyperkeratotic follicular papules on the arms and thighs that were usually associated with bilateral nipple hyperkeratosis. The incidence of these eruptions was 55% of patients with a mean time to onset of 32 days. The histopathological examination of the skin biopsy revealed pilar dystrophy and folliculitis [17]. Keratosis pilaris like eruptions presenting asymptomatic spinous hair follicle openings was seen during BRAFi treatment with a range of 6%–10% [11]. Sanlorenzo et al. reported that patients receiving dabrafenib developed keratosis pilaris (33.3%) more frequently than the vemurafenib group (16.7%) [14]. Topical steroid creams or exfoliants can be used for treatment [11]. Wang et al. also described a patient that developed diffuse folliculocentric papules with tiny keratotic plugs during vemurafenib treatment. They considered that this was a result of dysfunctional keratinocyte proliferation and treated the patient with ammonium lactate 12% cream [20].

Grade 1 and 2 hand-foot skin reactions were observed with the mean time to onset of 61 days in 60% of patients in the study by Boussemart et al. Hyperkeratotic, yellowish, and painful plaques were localized on the soles [17]. Lacouture et al. reported that palmar-plantar erythrodysesthesia occurred in 8%–10% of patients undergoing vemurafenib treatment. Topical moisturizers or keratolytic agents can be used for the treatment [11]. Hyperkeratosis as the most common cutaneous side effect was noted in 27% of patients in BREAK-2 [13]. Plantar, mucosal, vulvar, and gingival hyperkeratosis were also reported during BRAFi treatment [17, 19].

Boussemart et al. reported that, three weeks after the drug initiation, one patient developed greasy, scaly papules on the back with gingival lesions that indicated Darier's disease with distinctive histopathological findings [17]. Anforth et al. reported Grover's disease in 45% of patients treated with BRAF inhibitors longer than 52 weeks [19]. Chu et al. described Grover's disease such as a reaction with histopathological findings of acantholytic dyskeratosis during treatment of both BRAF inhibitors [21].

Cutaneous granulomatous eruption is a very rare side effect due to BRAFi therapy. Park et al. reported two cases of granulomatous reactions during BRAFi treatment. The first patient developed multiple erythematous and violaceous papules and erythematous indurated plaque after two months of dabrafenib and trametinib (MEK inhibitor) initiation. The lesions occurred on the areas of the metastatic subcutaneous disease. While the first biopsy revealed granulomatous inflammation with no melanoma cells, the second biopsy revealed granulomatous inflammation surrounding melanoma cells. It was speculated that the cause of the reaction was an immune response or activation against melanoma cells and indicated a positive therapeutic sign. The eruption resolved completely with clobetasol ointment use within two weeks. The second patient developed multiple erythematous, violaceous papules on his extremities after five months of vemurafenib treatment. The biopsy revealed granulomatous dermatitis with focal necrosis. The lesions disappeared spontaneously after the cessation of treatment and did not appear again after the resume of vemurafenib [22].

Garrido et al. reported that a patient developed erythematous nonpruritic plaques on his trunk and arm during dabrafenib and trametinib (MEK inhibitor) treatment. The histopathological findings of the first biopsy revealed granulomatous inflammation, admixed with melanophages. The second biopsy demonstrated granulomatous reaction. Atypical cells were not seen in either biopsy. The lesions improved spontaneously within few weeks [23].

Boussemart et al. described that 14% of patients developed panniculitis on the lower extremities. Lesions occurred with the mean time to onset of 78 days [17]. Sanlorenzo et al. found that the dabrafenib treatment group developed panniculitis more frequently than the vemurafenib treatment group, at rates of 33.3% and 16.7%, respectively [14]. Vasculitis, erythema nodosum were rarely seen AEs (<2%) [11].

Hair growth changes were observed in patients during BRAFi treatment such as cymotrichous, alopecia, or slower and thinner scalp hair growth [17, 19]. Alopecia was found in 11.1%–36% of patients in BRIM 2 and the study of Sanlorenzo et al., respectively [6, 14]. Boussemart et al. described alopecia with grade 1 or 2 in seven patients (16.6%) and thinner and slower scalp hair growth in 12 patients (28.5%) in their study [17]. Pigmentary changes such as vitiligo, nail changes, psoriasis flare, and urticaria were also seen during BRAFi treatment [18, 19, 24].

### 4.2. Photosensitivity

Photosensitivity reaction was one of the most reported adverse events related to BRAFi. The photosensitivity incidence was 52% in the BRIM 2 study, 7% with grade 2, and 1% with grade 3 in the BRIM 1 study of patients treated with vemurafenib [5, 6]. However, there was a broad range of percentages in several studies (22.2%–66.7%) [14, 18]. It was experienced more frequently in patients using vemurafenib during the summer time [17]. The cutaneous eruptions usually appeared on sun-exposed areas of the skin within hours of sun exposure, and in 3% of them with grade 3 or higher severity [6, 17, 25]. Photosensitivity developed within days of drug initiation and the median time to onset was 1.7 weeks [11]. Grade 1 cheilitis with a mean time to onset of 32 days, predominantly on the lower lip, was observed in patients with photosensitivity (14%). Facial erythematous eruption was reported with incidence of 17% and a mean time to onset of 62 days and mostly associated with photosensitivity [17]. Photosensitivity was less frequent for patients treated with dabrafenib compared to vemurafenib, so it can be used as an alternative treatment. Other skin lesions usually regressed after a couple of months, but photosensitivity could insist during treatment [26]. It was estimated that the cause of photosensitivity was dependent on the drug's chemical structure and ultraviolet A [27]. Broad spectrum sunscreens including UVA protection and protective clothing must be advised for all patients to avoid photosensitivity.

### 4.3. Malign and Benign Lesions

Both benign and malignant skin lesions occurred as side effects during BRAFi treatment. Cutaneous squamous cell carcinoma (cSCC) and keratoacanthoma (KA) were seen more frequently.

The incidence rate of cSCC or KA development was 22.2%–26% in Sanlorenzo et al.'s study and the BRIM 2 study, respectively [6, 14]. cSCC was reported in 12% of patients with grade 3 severity in the BRIM 3 study [8]. Cutaneous SCC incidence ranged from 16% to 26.7% in different studies [18, 19]. The mean time to first onset of cSCC was revealed as 7.1 weeks during vemurafenib treatment [11]. In the BREAK-1 study, cSCC occurred with a rate of 11% [12]. KAs were observed in 2% (grade 2) and 6% (grade 3) of patients in BRIM 3 study and 14% of patients in the study of Boussemart et al. [8, 17].

The underlying mechanism of cutaneous neoplasia was paradoxical activation of the MAPK pathway in sun-damaged skin cells with preexisting RAS mutations. BRAFi activated CRAF signaling in wild-type cells that induce ERK signaling, which promoted the development of cSCC [6, 8, 11]. The result of mutation analyses of resected cSCC lesions confirmed RAS mutations with a rate of 41% [11]. Su et al. reported that the prevalence of RAS mutations in patients treated with vemurafenib was 60%. The lesions tend to appear within the first weeks after vemurafenib initiation on sun-exposed areas of the body such as the face, neck, trunk, and thigh, which indicates that chronic sun exposure was a risk factor for cSCC development [11, 14, 17, 28]. Because of the early occurrence of the lesions, Su et al. suggested that vemurafenib may potentiate preexisting subclinical oncogenic lesions [28]. The treatment of cSCC and KA is simple surgical resection without dose interruptions or reductions [17]. KAs can regress spontaneously, as well [29].

Basal cell carcinoma was also observed in patients during treatment with both vemurafenib (13.3%) and dabrafenib (4%) [13, 18].

Actinic keratoses (AKs) were well-described lesions in patients during BRAFi treatment. Sanlorenzo et al. reported AKs in 44.4% of patients during vemurafenib treatment and 66.7% of patients during dabrafenib treatment [14]. Anforth et al. reported that 26% of patients developed AK after 52 weeks of BRAFi treatment, while Mattei et al. noted AK in 40% of patients in their study [18, 19]. These lesions are known as precursors of cSCC; therefore, surgical removal must be completed as soon as possible [18].

Changes in melanocytic lesions were seen in patients with advanced melanoma during treatment with BRAF inhibitors [30]. These changes were seen as involution of nevi, changes of nevi color, and size and development of new melanoma from preexisting nevi and also new primary melanoma [31]. Anforth et al. reported changes in nevi in 11% of patients in the course of BRAFi therapy. While changes in size and color of melanocytic nevi such as hyperpigmentation and regression and the development of new nevi occurred in their study, no new primary melanomas were observed [19]. Haenssle et al. described a patient who developed involution of preexisting melanocytic nevi during vemurafenib treatment. This situation occurred because the nevi also harbored the BRAF V600E mutation and were affected by the treatment. They also performed a biopsy on preexisting nevi with wild-type BRAF, which showed increased pigmentation and size. The findings demonstrated cytologic dysplasia, melanophages, and lymphohistiocytic inflammation [32].

Zimmer et al. analyzed 22 cutaneous melanocytic lesions in 19 patients with metastatic melanoma, undergoing treatment with selective BRAF inhibitors. Seven invasive and five in situ melanomas developed in 11 patients with a mean time of eight weeks. None of these 12 new primary melanomas harbored the BRAF V600 mutation. Five of these new melanomas appeared on sun-exposed areas. Nine of ten preexisting nevi were classified as dysplastic nevi and one lesion was classified as a common nevus. The mean time of change was 17.5 weeks [30]. Dalle et al. reported that primary melanomas occurred four to twelve weeks after vemurafenib initiation. These melanomas were BRAF wild-type and diagnosed from atypical melanocytic lesions [33].

Anforth et al. reported a patient who developed eruptive nevi during the treatment of a new selective BRAF inhibitor LGX818. Multiple lesions occurred on the back, chest, and leg, two months after the treatment. The histopathological examination revealed pigmented compound nevus [34].

Sanlorenzo et al. observed warts in the course of BRAFi treatment in 22.2% of patients [14]. Anforth et al. reported verruca vulgaris in 5% of patients after 52 weeks of treatment with BRAFi. The histopathological examination of these lesions confirmed viral inclusions such as koilocytes and keratohyalin granules [19]. Mattei et al. reported that 46.7% of patients developed warts with a median time of four weeks in their study [18]. Verrucal keratoses were seen in patients during BRAFi therapy. These premalignant papules were precursors to cutaneous SCC and were reported in 18% of patients with BRAFi treatment longer than 52 weeks in the study of Anforth et al. [19]. Boussemart et al. described polypoid lesions with a hyperkeratotic surface as benign verrucous papillomas on the face, limbs, and trunk during vemurafenib treatment. They occurred with a mean time to onset of 35 days in 79% of patients. It was the most common cutaneous side effect in their study [17].

Mattei et al. reported 26.7% of patients developed milia and one patient developed infundibular occlusion cysts [18]. Boussemart et al. also observed milia cysts on the face of the 31% of patients, which occurred in a range of 21–83 days. Epidermoid cysts were also reported in 33% of patients with a mean time to onset of 108 days in their study [17]. Gebhardt et al. published a single case of a patient that developed multiple, asymptomatic superficial keratinous cysts presenting milia. The lesions occurred on sun-damaged areas within seven weeks after vemurafenib initiation and disappeared after discontinuation [35]. Treatment is unnecessary unless physical symptoms or cosmetic concerns insist. Houriet et al. reported a patient who developed localized epidermal cysts during combined treatment with vemurafenib and radiotherapy. Lesions appeared on previously irradiated localized areas after two months of vemurafenib initiation. It was estimated that the cause was radiosensitivity [36]. It was advised that vemurafenib should be interrupted seven days before and after radiotherapy [37].

Garrido et al. described a patient that developed multiple nodular lesions on his back during vemurafenib treatment for advanced melanoma. The lesions occurred after four months of treatment. The biopsy was performed and histopathological findings revealed primary, cutaneous small/medium CD4 T-cell lymphoma. The authors believed that the activation of immunity that was induced by vemurafenib was the cause of this situation [38].

## 5. Conclusion

BRAF inhibitors have a crucial role in patients with inoperable metastatic melanoma. They have significant benefits in the prognosis, but some cutaneous adverse reactions can occur in their clinical use. The combination therapies with MEK inhibitors reduce the side effects that occur with monotherapy alone. The patients undergoing BRAFi treatment should be examined in the course of the treatment periodically by selective dermatologic working groups. Early diagnosis and treatment of these cutaneous side effects can improve the patient's quality of life.

## Figures and Tables

**Table 1:** Percentage of common (>5%) cutaneous adverse events with vemurafenib treatment.

Adverse events	Percentage (%)
Verrucous papilloma/wart	22.2 [14]–79 [17]
Rash^*∗*^	64–71 [11]
Photosensitivity	22.2 [14]–66.7 [18]
Hand-foot skin reaction (PPD)	5.6 [14]–60 [17]
Hair growth modification	45 [17]
Actinic keratosis	40 [18]–44.4 [14]
Alopecia	11.1 [14]–36 [6]
Pruritus	29 [6]–33.3 [14]
Xerosis	11.1 [14]–33 [17]
Milia	26.7 [18]–31 [17]
cSCC and KA	22.2 [14]–26.7 [18]
Panniculitis	14 [17]–16.7 [14]
Keratosis pilaris	16.7 [14]
Cheilitis	14 [17]
BCC	13.3 [18]
Nipple hyperkeratosis	12 [17]
Nevi changes	5.6 [14]–10 [17]

PPD: palmar-plantar dysesthesia; cSCC: cutaneous squamous cell carcinoma; KA: keratoacanthoma; BCC: basal cell carcinoma.

^*∗*^In various studies, rash was categorised as erythema, maculopapular rash, folliculitis, and not otherwise specified; however some authors pointed out rash as a general term.

Indicated numbers beside the percentages denote the related references.

**Table 2:** Percentage of common (>5%) cutaneous adverse events with dabrafenib treatment.

Adverse events	Percentage (%)
Actinic keratosis	10.7 [9]–66.7 [14]
Hyperkeratosis	27 [13]–39.4 [9]
Pruritus	5.35 [9]–33.3 [14]
Photosensitivity	2.67 [9]–33.3 [14]
Panniculitis	33.3 [14]
Keratosis pilaris	33.3 [14]
Alopecia	28.8 [9]
Skin papilloma	15 [13]–25.13 [9]
Palmar-plantar dysesthesia	20.32 [9]
Rash	18.72 [9]
Dry skin	10.7 [9]
Seborrheic keratosis	8.56 [9]
Hair texture abnormal	6.42 [9]
cSCC	1.6 [9]

cSCC: cutaneous squamous cell carcinoma.

Indicated numbers beside the percentages denote the related references.
